# Ex Vivo Immune Function and Modulatory Effects of Calcitriol in Dogs with Naturally Occurring Diabetes Mellitus

**DOI:** 10.3390/vetsci11050193

**Published:** 2024-04-28

**Authors:** Jared A. Jaffey, Rachael Kreisler, Thomas K. Graves, Layla Al-Nakkash, Robert C. Backus, Lauren Allison

**Affiliations:** 1 Department of Specialty Medicine, Midwestern University, College of Veterinary Medicine, Glendale, AZ 85308, USA; t.graves@midwestern.edu (T.K.G.); lallison.dvm@gmail.com (L.A.); 2 Department of Pathology and Population Medicine, Midwestern University, College of Veterinary Medicine, Glendale, AZ 85308, USA; rkreis@midwestern.edu; 3 Department of Physiology, Midwestern University, College of Graduate Studies, Glendale, AZ 85308, USA; lalnak@midwestern.edu; 4 Department of Veterinary Medicine and Surgery, University of Missouri, College of Veterinary Medicine, Columbia, MO 65211, USA; backusr@missouri.edu

**Keywords:** vitamin D, type 1 diabetes mellitus, whole blood culture, flow cytometry, inflammation, canine

## Abstract

**Simple Summary:**

Naturally occurring diabetes mellitus (NODM) is one of the most common endocrine disorders in dogs and is most similar to type 1 diabetes mellitus (T1DM) in human patients. Immune responses in people with T1DM are abnormal and may contribute to a variety of long-term complications. There is very little information regarding immune function in dogs with NODM. Vitamin D deficiency is a relatively frequent finding in diabetic human patients. This is important because vitamin D has several positive effects on the immune system. Therefore, it is unsurprising that some studies demonstrate that vitamin D supplementation is beneficial in people with T1DM. While vitamin D interacts with the immune system in dogs, there have been no studies evaluating its effect in diabetic dogs. Therefore, our study sought to evaluate several components of immune function and the modulatory effects of calcitriol in diabetic dogs. We found that like human patients with T1DM, the diabetic state causes cytokine and phagocytic dysregulation in dogs. Vitamin D affected leukocyte cytokine secretion but not phagocytosis. The level of clinical control in diabetic dogs did not affect the immune function variables investigated in this study.

**Abstract:**

Human patients with type 1 diabetes mellitus (T1DM) are susceptible to several long-term complications that are related to glycemic control and immune dysregulation. Immune function remains relatively unexplored in dogs with naturally occurring diabetes mellitus (NODM). Calcitriol improves various aspects of immune function in a variety of species, but its effect in diabetic dogs remains unexplored. Therefore, the objectives of this study were to (i) evaluate immune function in dogs with NODM and determine if differences exist based on the level of clinical control and (ii) assess the immunomodulatory effects of calcitriol. Twenty diabetic dogs (clinically controlled, n = ten, not controlled, n = ten) and 20 non-diabetic, healthy control dogs were included in this prospective, case–control study. Whole blood was incubated with calcitriol (10^−7^ M) or negative control, after which the samples were divided for phagocytosis and leukocyte cytokine response experiments. The phagocytosis of opsonized *Escherichia coli* (*E. coli*) was evaluated with flow cytometry. The samples for leukocyte cytokine response evaluations were stimulated with lipopolysaccharide (LPS), lipoteichoic acid (LTA), or phosphate buffer solution (PBS; negative control), and tumor necrosis factor (TNF)-α, interleukin (IL)-6, IL-8, and IL-10 were measured in supernatant using a canine-specific multiplex bead-based assay. The leukocytes from diabetic dogs produced higher concentrations of IL-10 (*p* = 0.01), IL-6 (*p* < 0.0001), and IL-8 (*p* < 0.0001) than the control dogs while controlling for the intervention and stimulant. Calcitriol decreased the supernatant concentrations of TNF-α (*p* < 0.001) and IL-8 (*p* = 0.04) with concomitant increases in IL-6 (*p* = 0.005). Diabetic dogs had a lower percentage of leukocytes undergoing phagocytosis (*p* < 0.0001) but a higher number of bacteria phagocytized per cell (*p* = 0.001) when compared to the control dogs. Calcitriol had no effect on phagocytic capacity. Lastly, the status of clinical control in diabetic dogs did not yield differences in immune function. These results support that dogs with NODM exhibit immune dysregulation and warrant additional investigation.

## 1. Introduction

Diabetes mellitus is a common endocrinopathy in dogs with an estimated prevalence in pet populations that range from 0.2 to 1.3% [1,2,3,4,5,6,7,8,9]. A recent report estimated that 165,000 pet dogs in the United States have diabetes mellitus [10]. The pathogenesis of diabetes mellitus in dogs can vary; however, the most common clinically recognized form parallels type 1 diabetes mellitus (T1DM) in human patients [11]. Regardless of the underlying etiology, dogs with naturally occurring diabetes mellitus (NODM) commonly demonstrate one or more condition of polyuria, polydipsia, polyphagia, and weight loss because of protracted hyperglycemia and glucosuria [12].

Diabetic complications in people, and likely dogs, are related to chronic hyperglycemia and its downstream effects on contributing to cytokine dysregulation [13,14,15,16,17,18] and immune dysfunction [19,20,21,22,23,24]. Moreover, a pro-inflammatory state in human patients with T1DM has been linked to poor glycemic control [15,17,25,26]. Pro-inflammatory cytokines such as tumor necrosis factor (TNF)-α contribute to insulin resistance by inhibiting the insulin receptor tyrosine kinase activity and downregulating cellular glucose transporter genes [27,28]. In addition, increased TNF-α and interleukin (IL)-6 levels can lead to exaggerated leukocyte activation and tissue damage, leading to impaired mucosal integrity and subsequent infection [13]. There is abundant evidence highlighting the dysfunction of innate immunity in humans with T1DM. Neutrophils from humans with T1DM show derangements in almost all functions including migration, adhesion, the release of lytic proteases, phagocytosis, killing capacity, and apoptosis [19,20,21,22]. Monocytes from diabetic patients also exhibit abnormal functions [20,23,24]. There is a fraction of available information regarding the cytokine profile and immune function in diabetic dogs [29,30,31]. Expanding the paucity of literature related to the inflammatory milieu and immune function in dogs with NODM is important to improving our understanding of this common endocrinopathy.

The active metabolite of vitamin D calcitriol improves several aspects of innate immune function in many different species, including dogs, such as increased antimicrobial peptides, microbial phagocytic and killing capacities, and the modulation of exaggerated proinflammatory cytokine responses [32,33,34,35,36,37,38,39,40]. Vitamin D deficiency is associated with poor glycemic control and the development of various complications in people with T1DM [41,42,43,44,45]. Taken together, it is unsurprising that adjunctive vitamin D supplementation in human patients with T1DM has been shown in some studies to improve glycemic control and the risk for diabetes-related complications [46,47,48]. More information regarding the immunologic effects of calcitriol in diabetic dogs is needed, as evidence in people with T1DM suggests that adjunctive vitamin D supplementation may have similar beneficial effects.

This prospective case–control study had three objectives (i) to compare the stimulated leukocyte cytokine production and granulocyte/monocyte (GM) phagocytic capacity of *Escherichia coli* (*E. coli*) in diabetic dogs and non-diabetic healthy controls, (ii) to determine the effect that calcitriol has on leukocyte cytokine production and GM phagocytic capacity, and (iii) assess whether the clinical control of diabetes mellitus affects these immune function variables. We hypothesized that diabetic dogs would have different leukocyte cytokine responses and phagocytic capacity compared with non-diabetic healthy controls. Furthermore, we hypothesized that calcitriol and clinical control status of diabetes mellitus would affect one or more of these immune function variables.

## 2. Materials and Methods

### 2.1. Criteria for Selection of Dogs and Study Design

Client-owned dogs with NODM treated with ≥0.25 units/kg of insulin administered once every 12 h and non-diabetic controls were prospectively identified by a combination of emailing a recruitment flyer to primary care veterinarians in our geographic region and an interrogation of the electronic medical records system at the Companion Animal Clinic at Midwestern University College of Veterinary Medicine. Diabetic dogs were classified as having clinically controlled diabetes mellitus if the dog exhibited no polyuria, polydipsia, or polyphagia and there had been no insulin dose adjustments within 4 weeks of enrollment. Dogs were excluded if they were obese or had received vaccinations within 1 month of enrollment. Dogs with relevant comorbidities or concurrent illness within 60 days of enrollment were also excluded. A board-certified small animal internist (JAJ) determined whether a comorbid condition was clinically relevant. A second population of health-, age- (i.e., ±2 years), breed-, and sex-matched non-diabetic healthy control dogs were enrolled. Control dogs were included after a review of their clinical history, physical examination, complete blood count, and serum chemistry by a single investigator (JAJ). Control dogs were enrolled if they were non-obese, had no illnesses within 6 months of enrollment, and no vaccinations within 1 month of enrollment. Informed written consent was obtained for all dogs. This study was conducted in accordance with guidelines for clinical studies and approved by the Midwestern University Animal Care and Use Committee (protocol: #2944; approval date: 14 June 2019).

### 2.2. Data and Sample Collection

Medical records were reviewed for each dog enrolled. The age, sex, weight, body condition score (BCS), and breed were recorded for each. Other relevant details were recorded when indicated, such as maintenance diet information and insulin type and dosage. Hematology, serum biochemistry, serum fructosamine, and urinalysis tests were measured at a commercial laboratory (Antech Diagnostics, USA).

### 2.3. Calcitriol

Calcitriol (Sigma-Aldrich, St. Louis, MO, USA) was dissolved in 75% ethanol (Sigma-Aldrich, St. Louis, MO, USA) to make a stock solution of calcitriol at 24 nmol/mL and stored light-protected at 4 °C as previously described [40].

### 2.4. Blood Sample Collection and Calcitriol Treatment

A blood sample (6 mL) was collected from each dog into tubes containing lithium heparin as an anticoagulant and processed within 1 h. Blood (3 mL) was allocated into 2 separate conical tubes and diluted 1:2 with RPMI 1640 culture medium (Thermo Fisher Scientific, Carlsbad, CA, USA) containing 200 U of penicillin/mL and 200 mg of streptomycin/mL. The blood–RPMI mixture was then incubated with calcitriol (final concentration, 10^−7^ M) or ethanol negative control diluent for 24 h at 37 °C in 5% CO_2_ in the dark as previously described [40].

### 2.5. Leukocyte Cytokine Production

After incubation with calcitriol or ethanol for 24 h, samples from the conical tubes were transferred to 96-well plates and stimulated with lipopolysaccharide (LPS) from *Escherichia coli* O127:B8 (final concentration, 100 ng/mL, Sigma Aldrich, St Louis, MO, USA), lipoteichoic acid (LTA) from *Streptococcus faecalis* (final concentration, 1 µg/mL, Sigma-Aldrich, St Louis, MO, USA), or a phosphate-buffered saline (PBS) control substance. Plates were incubated for 24 h at 37 °C in 5% CO_2_ in the dark. Following incubation, plates were centrifuged (400× *g* for 7 min) at 21 °C as previously described [40]. The supernatant was collected and stored at −80° C for batch analysis. For the analysis, samples were thawed, and then TNF-α, IL-6, IL-8, and IL-10 were measured in supernatant with a previously validated canine cytokine-specific multiplex bead-based assay (Milliplex MAP canine cytokine–chemokine panel, EMD Millipore Corp, Billerica, MA, USA). The median fluorescence intensity and cytokine concentration in each sample was measured in duplicate with the appropriate controls and associated data analysis software (Milliplex Analyst version 5.1, EMD Millipore Corp, Billerica, MA, USA). The lower limit of detection for TNF-α, IL-10, and IL-6 was 48.8 pg/mL, and for IL-8, it was 195 pg/mL.

### 2.6. Phagocytosis of E. coli

Phagocytic capacity was determined with a commercially available assay (PhagoTest, Orpegen Pharma, Heidelberg, Germany), validated for use in canines. The blood mixture that had been incubated with either calcitriol or ethanol for 24 h was incubated with FITC-labeled, opsonized *E. coli* strain LE392. The control samples were incubated on ice for 10 min while test samples were incubated in a 37 °C water bath for 10 min. Phagocytosis was then arrested with test samples being placed on ice, and a quenching solution was added to extinguish surface-bound FITC-labeled *E. coli*. The cells were then washed, the erythrocytes lysed, and the cells washed again before a DNA stain (propidium iodide) was added to facilitate the exclusion of aggregated artifacts of bacteria or cellular debris without intact DNA.

### 2.7. Flow Cytometry

Flow cytometry was performed at the Midwestern University College of Veterinary Medicine Immunology Laboratory using a Guava easyCyte 12HT (Luminex Corporation, Austin, TX, USA) and associated data analysis software (Guava-Soft 3.2, Luminex Corporation, Austin, TX, USA). A minimum of 20,000 events per sample were recorded. The gating scheme has been previously reported [40]. Briefly, forward scatter height (FSC-H) vs. side scatter height (SSC-H) cell size and granularity were used to define the primary population of interest (GM). To further eliminate non-viable cells, DNA-positive staining cells were gated and then applied to a histogram to determine the percentage of FITC-positive cells and the mean fluorescent intensity. Phagocytic capacity was noted as (i) the percentage of GM cells that had internalized FITC-labeled *E. coli* and (ii) the mean fluorescent intensity (MFI), a method of quantifying the number phagocytosed bacteria per cell.

### 2.8. Statistical Analysis

Statistical analysis was performed by commercial software (SigmaPlot, Systat Software version 14.5, and Stata Statistical Software version 18, StataCorp LLC, College Station, TX, USA). Non-normally distributed continuous data were described as the median and interquartile range (IQR). Continuous data with a normal distribution were presented as the mean and standard deviation (SD). When the measured cytokine concentrations fell below the lower limit of detection, data were recorded at the lower limit of detection for statistical purposes. The relationship between leukocyte cytokine expression, diabetic status, and exposure type (i.e., calcitriol or ethanol) was assessed for each cytokine via multilevel mixed-effects generalized linear regression (MMEGLR) controlling for the stimulant with dog as a random effect and using robust standard errors [49]. The relationships between phagocytic capacity and diabetic status and controlled versus uncontrolled diabetic status were similarly assessed. Models were built using forward selection and biological plausibility. The model fit was assessed through standardized residuals and competing models compared using Akaike information criterion (AIC)/Bayesian information criterion (BIC) values. A *p*-value of <0.05 was considered significant. Data from this study are available at https://www.kaggle.com/datasets/jaredjaffey/canine-nodm-immune-and-calcitriol accessed on 22 April 2024.

## 3. Results

### 3.1. Animal Population

Forty-one dogs were eligible for inclusion in this prospective case–control study. One dog was excluded because an appropriately matched control dog was not identified, leaving 40 dogs (NODM, n = 20; controls, n = 20). The demographic information is presented in Table 1. Diabetic dogs were maintained on either Neutral Protamine Hagedorn (NPH) (55%, 11/20) or porcine lente (45%, 9/20) insulin. The median dosage of insulin administered once every 12 h to NODM dogs was 0.75 units/kg (IQR, 0.40–0.88; range, 0.3–1.2 units/kg). All dogs were fed a commercially available pet food. Ten dogs each were clinically controlled or uncontrolled. The diabetic dogs had greater serum glucose concentrations (median, IQR; 317 mg/dL, 237–501.3) than the control dogs (101 mg/dL, 90.5–106; *p* < 0.001). Similarly, the diabetic dogs had greater serum fructosamine concentrations (median, IQR; 491 mg/dL, 433.3–603) than the control dogs (246.5 mg/dL, 233–278.5; *p* < 0.001). 

### 3.2. Leukocyte Cytokine Responses

**Figure 1:**
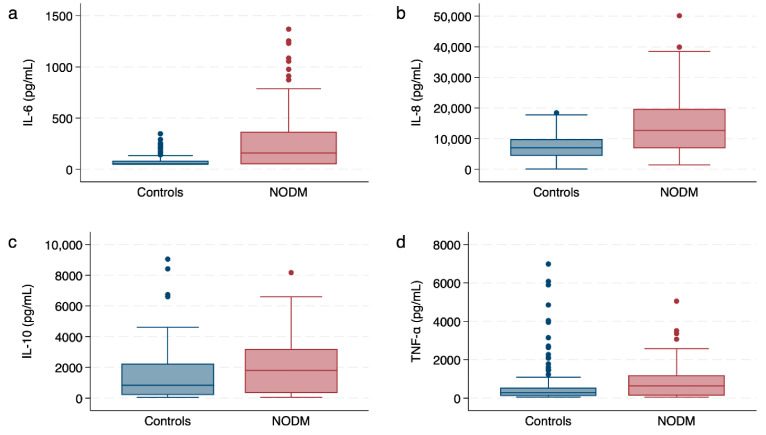
Box and whisker plots illustrating leukocyte cytokine response for (**a**) IL-6, (**b**) IL-8, (**c**) IL-10, and (**d**) TNF-α between control dogs and dogs with naturally occurring diabetes mellitus (NODM), controlling for intervention (calcitriol or ethanol) or stimulant (phosphate-buffered solution, lipopolysaccharide, lipoteichoic acid). Each of the 40 dogs, 20 per group (control or NODM), had a cytokine value for each of the six unique combinations of intervention and stimulant for a total of 120 values per group for each plot. Line at median, bounds of box at the 25th and 75th percentile, whiskers at the upper and lower adjacent values (Tukey method), and dots at outliers beyond the adjacent values.

The 20 dogs from each group had leukocyte cytokine values generated for the six combinations of intervention (calcitriol or ethanol) and stimulant (PBS, LPS, LTA), resulting in 120 values for each type of leukocyte cytokine for each group (controls or NODM) (Figure 1, Supplementary Table S1). The median supernatant concentration for IL-6 was 49 pg/mL (IQR, 49–85; range, 48–348 pg/mL) for the controls and 106 pg/mL (IQR, 49–368; range, 49–1369 pg/mL) for the diabetic dogs. In MMEGLR (Poisson family), IL-6 was 216% greater for the diabetic dogs compared to the controls (*p* < 0.0001; 95% CI, 127–340) controlling for stimulant and intervention (Table 2). In a similar model containing only diabetic dogs, there was no difference (*p* = 0.53) in IL-6 based on the status of the clinical control as the uncontrolled diabetics had a median of 168 pg/mL (IQR, 49–508; range, 49–1369) and the controlled diabetics had a median of 138 pg/mL (IQR 49–298; range 49–1255).

For IL-8, the median supernatant concentration was 7042 pg/mL (IQR, 4369–9911; range, 88–18,442 pg/mL) for the controls and 12,724 pg/mL (IQR, 6869–19,698; range, 1430–50,122) for the diabetic dogs. Interluekin-8 concentrations were predicted to be 95% greater for the diabetic dogs (*p* < 0.0001; 95% CI, 40–172) using MMEGLR (negative binomial family). There was no difference in IL-8 for dogs with uncontrolled versus controlled diabetes (*p* = 0.19), with a median of 10,505 pg/mL (IQR, 5573–18,244; range, 2828–28,210 pg/mL) and 16,526 pg/mL (IQR, 9981–22,730; range, 1430–50,122), respectively.

The median supernatant concentration for IL-10 was 829 pg/mL (IQR, 200–2243; range, 49–9044 pg/mL) for controls and 1802 pg/mL (IQR, 328–3205; range, 49–8170 pg/mL) for diabetic dogs. In MMEGLR (negative binomial family), IL-10 concentrations were predicted to be 60% greater for diabetic dogs (*p* = 0.01; 95% CI, 10–133) after excluding two outliers with Pearson residuals greater than 4 (both values from the control dogs with PBS incubation, one in calcitriol and one in ethanol). Although calcitriol was not significant in the model (Table 2), the AIC and BIC values were lower for the model retaining calcitriol, and the coefficient and *p* values did not change appreciably. The *p* value for diabetic status was 0.09 without exclusion of the outliers, and the changes to the coefficients and standard errors were less than 10%. Dogs with uncontrolled diabetes did not have a different supernatant concentration of IL-10 compared to dogs with controlled diabetes (*p* = 0.91), with a median of 1986 pg/mL (IQR, 328–3205; range, 49–6270 pg/mL) and 1537 pg/mL (IQR, 360–3206; range, 49–8170 pg/mL), respectively.

Tumor necrosis factor-α had a median supernatant concentration of 280 pg/mL (IQR, 106–546; range, 49–6981 pg/mL) for controls and 636 pg/mL (IQR, 124–1192; range, 48–5048 pg/mL) for diabetic dogs. Tumor necrosis factor-α concentrations were not different between the diabetic and control dogs (*p* = 0.06; 95% CI, −1 to 127) after excluding the same outliers as for IL-10 using MMEGLR (negative binomial family). The *p* value for TNF-α without excluding the outliers was 0.23. No difference was found for the TNF-α levels (*p* = 0.86) between dogs with uncontrolled diabetes, with a median of 594 pg/mL (IQR, 107–1210; range, 49–3510 pg/mL), and controlled with a median of 677 pg/mL (IQR, 128–1164; range, 48–5048 pg/mL).

### 3.3. Effect of Calcitriol

In the same multivariable mixed-effects linear regression model (Table 2), incubation in calcitriol was found to increase IL-6 concentrations by 42% (*p* = 0.005; 95% CI, 11 to 81) compared to incubation in ethanol while controlling for the stimulant and group. Interleukin-8 concentrations decreased by 7% (*p* = 0.04; 95% CI, −14 to 0), TNF-α concentrations decreased by 33% (*p* < 0.001; 95% CI, −44 to −20), and IL-10 (*p* = 0.11) was not different.

### 3.4. Phagocytic Capacity of Opsonized-E. coli

**Figure 2:**
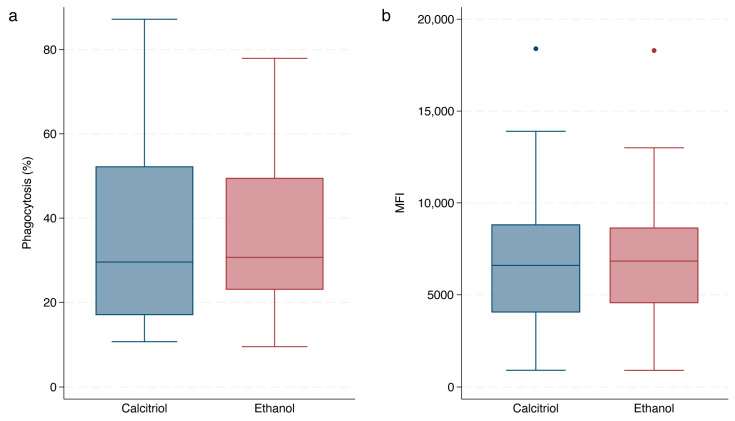
Box and whisker plots illustrating (**a**) percentage of granulocytes and monocytes (GM) phagocytizing opsonized *E. coli* and (**b**) the average number of *E. coli* phagocytized per cell between dogs with naturally occurring diabetes mellitus (NODM) and controls, irrespective of intervention (calcitriol or ethanol). Each of the 40 dogs, 20 per group (control or NODM), had a measure of phagocytosis for each intervention for a total of 40 values per group for each plot. Line at median, bounds of box at the 25th and 75th percentile, whiskers at the upper and lower adjacent values (Tukey method), and dots at outliers beyond the adjacent values.

The phagocytic capacity of opsonized *E. coli* was assessed at 24 h via percent phagocytosis and MFI (i.e., average number of *E. coli* phagocytized per cell) for both the ethanol and calcitriol interventions for the 20 dogs in each group (Figure 2). In MMEGLR (Poisson family), diabetic dogs had a 47% decrease (95% CI, −60 to −30; *p* < 0.0001) in the percentage of cells phagocytizing *E. coli* (median, IQR; 23%, 16–29) compared to control dogs (48%, 31–61). Diabetic dogs had 76% more phagocytized *E. coli* per cell (median, IQR; 8539 bacteria/cell, 6572–10,400) compared to control dogs (5318 bacteria/cell, 3370–6900), irrespective of the intervention (*p* = 0.001) in MMEGLR (negative binomial family). There was no difference in the percentage of cells phagocytizing *E. coli* (*p* = 0.3) or the number of bacteria phagocytized per cell (*p* = 0.65) between the calcitriol and ethanol interventions, irrespective of the group (Supplemental Figure S1), nor was there any difference in the percentage of cells phagocytizing *E. coli* (*p* = 0.71) or the number of bacteria phagocytized per cell (*p* = 0.82) between uncontrolled and controlled diabetes, irrespective of the intervention (Supplemental Figure S2).

## 4. Discussion

This prospective case–control study explored several aspects of immune function and the ensuing modulatory effects of calcitriol in dogs with NODM. Moreover, subanalyses were performed to assess whether the level of diabetic clinical control affected immune function. We found that leukocytes from diabetic dogs produced higher concentrations of IL-10, IL-6, and IL-8 in supernatant, controlling for the intervention (i.e., calcitriol or ethanol) and stimulant (i.e., PBS, LPS, or LTA). Next, we found that calcitriol had no differential effects on leukocyte cytokine responses in the diabetic and control dogs, regardless of stimulant exposure. However, supernatant concentrations of IL-8 and TNF-α were decreased, while IL-6 increased with the incubation of calcitriol while controlling for the stimulant and group. Diabetic dogs exhibited an abnormal phagocytosis of opsonized *E. coli* characterized by a decreased percentage of leukocytes performing phagocytosis with a concomitant increase in the number of organisms phagocytized per cell. Lastly, the status of the diabetic clinical control did not yield differences in immune function.

Leukocytes from diabetic dogs produced higher concentrations of IL-10, IL-6, and IL-8 in supernatant than the controls. The supernatant concentrations of TNF-α were higher in diabetic dogs, but this difference was not statistically significant (*p* = 0.06). These results reinforce that the diabetic state in dogs, similar to people, elicits cytokine dysregulation [13,14,15,16,18,25,29,30,44]. The prevailing theory used to explain this phenomenon is the progressive accumulation of endogenous advanced glycation end products (AGEs) via the Maillard reaction that occurs as a byproduct of chronic hyperglycemia [50]. Advanced glycation end products exert their deleterious effects through direct irreversible damage to proteins, which intensify reactive oxygen species (ROS) formation, stimulate pro-inflammatory events, and alter intracellular signaling [51,52,53]. Diabetic dogs have been shown to have increased plasma AGEs compared to control dogs, and plasma AGEs had a moderate positive correlation with blood glucose concentrations [54]. Cytokines have been implicated in the development of many long-term complications in diabetic human patients and may have a similar nefarious role in dogs with NODM. As such, additional research focused on cytokine dysregulation in diabetic dogs is warranted.

There was no difference in cytokine concentrations in supernatant from diabetic dogs that were clinically controlled versus those that were not. These results are in contrast to our hypothesis and are dissimilar to previous studies in diabetic people [14,15,17,25]. One possible explanation for our contrasting results is the criteria used to classify diabetic control. We stratified dogs based on the level of clinical control rather than using a surrogate marker for glycemic control, as was conducted in some human studies [15,25]. Fructosamine are glycated proteins in blood that have historically been used to monitor glycemic control in diabetic dogs [55]. However, recent research has highlighted the inadequacy of serum fructosamine to predict glycemic control in diabetic dogs [56,57,58]. Kulseng et al.’s [14] study in (1996) measured cytokines in supernatant after the antigen stimulation of peripheral blood mononuclear cells in people with T1DM at the time of diagnosis and again 3 months later after the glycemic control improved. The results from that study demonstrated that antigen-stimulated TNF-α production in supernatant decreased over time in patients as the glycemic control improved [14]. A future similarly designed longitudinal study in diabetic dogs would circumvent the inaccuracy of a single serum fructosamine result to dictate glycemic control.

The incubation of peripheral blood leukocytes with calcitriol, while controlling for the stimulant and group (i.e., controls or NODM), resulted in decreased supernatant concentrations of TNF-α and IL-8 along with increases in IL-6. There were no differences in cytokine concentrations between diabetic dogs and controls or based on the clinical control status in diabetic dogs. Calcitriol directly and indirectly blocks the transcription of NF-κβ and the MAPK-mediated production of pro-inflammatory cytokines (e.g., IL-1β, TNF-α, IL-6, IL-8) in various cell types from people and mice [59,60,61,62,63,64,65,66]. Several in vitro studies have demonstrated that the incubation of peripheral blood leukocytes with calcitriol decreases supernatant concentrations of TNF-α in dogs [38,39,40,67]. To date, no canine studies have identified an effect of calcitriol on the leukocyte production of IL-6 [38,68], and investigations focused on IL-8 are lacking. The finding of increased supernatant concentrations of IL-6 was unexpected and cannot be reasonably explained from our data. These results contradict in vivo and ex vivo studies in people that demonstrate the consistent downregulation of IL-6 by calcitriol [69,70,71].

Diabetic dogs displayed a dysregulated phagocytic function of opsonized *E. coli* characterized by a decreased percentage of leukocytes performing phagocytosis with a concomitant increase in the number of bacteria phagocytized per cell. The overall net effect on phagocytic function is unknown. The level of diabetic clinical control had no effect on phagocytosis. No other studies have investigated phagocytic capacity in dogs with NODM; however, leukocyte microbicidal activity is impaired in human patients with T1DM and non-obese diabetic mice [21,72,73,74]. Studies in rabbits highlight that hyperglycemia impairs phagocytosis in both neutrophils and monocytes, and insulin administration reverses this dysfunction [75,76]. Our study focused on a singular pathway of phagocytic capacity in an otherwise expansive and coordinated system of pathogen recognition, signaling, and internalization. Therefore, the results from the study herein should not be interpreted as a comprehensive evaluation of phagocytosis in dogs with NODM. Additional studies that interrogate a variety of immune cell pathogen recognition and killing pathways are needed to better understand phagocytic function in diabetic dogs.

The leukocyte phagocytic capacity of opsonized *E. coli* was not affected by incubation with calcitriol in this study. Calcitriol augments the phagocytosis of immune cells from people, rats, and cows [34,37,77,78]. However, two previous studies in dogs failed to show that calcitriol affected the GM phagocytic capacity of *E. coli* [40,68]. One possible reason for these conflicting results is that the dog studies focused on the phagocytosis of *E. coli*, whereas the previously mentioned studies in other species used *Mycobacterium bovis*, heat-killed baker’s yeast, fluorescent carboxyl microspheres, and *Pseudomonas aeruginosa* [34,37,77,78]. There are conflicting reports on the benefit of calcitriol against *E. coli.* [79,80]. Therefore, it is possible that calcitriol induces variable host immune responses dependent on the encountered pathogen.

Our study had several limitations. We utilized whole blood cultures to evaluate various aspects of immune function and the modulatory effects of calcitriol. These methodologies were purposefully expansive to allow cellular reactions to occur in a more physiologic milieu. This approach may improve the translational relevance of the in vitro results. With that said, different results may have been identified if immune function testing was assessed in specific cell types. We investigated phagocytic function in the context of leukocyte interactions with *E. coli* alone, and thus, our results do not provide a comprehensive outlook on phagocytosis in dogs with NODM. Our investigation focused on supernatant concentrations of TNF-α, IL-6, IL-10, and IL-8 because these cytokines are most commonly implicated as being aberrant in humans with T1DM [13,14,15,16,18,25,44]. However, a more expansive profile of cytokines must be evaluated in order to determine whether diabetic dogs have a specific cytokine immunosignature. Diabetic dogs were stratified into subgroups based on the level of clinical control, which is not necessarily always congruent with glycemic control. This was an imperfect solution in an exploratory study to the lack of an accurate and reliable surrogate marker of glycemic control in diabetic dogs.

## 5. Conclusions

In conclusion, our study provides evidence that diabetic dogs exhibit cytokine and phagocytic dysregulation, which is not affected by the status of clinical regulation. Calcitriol altered leukocyte cytokine production without differential effects based on whether a dog had diabetes or not. These results provide a foundation for the further refinement and development of studies to assess immune responses and modulation by calcitriol in dogs with NODM.

## Figures and Tables

**Table 1:** Descriptive characteristics in dogs with naturally occurring diabetes mellitus and non-diabetic healthy controls.

Variable	NODM	Control	*p*-Value
Number of dogs	20	20	---
Age (years) ^a^	9.3 (1.9)	9.5 (1.9)	0.78 ^c^
Weight (kgs) ^b^	7.6 (6.4)	8.4 (18.1)	0.59 ^d^
BCS ^b^	5 (1)	5 (1)	0.64 ^d^
Sex (female, male)	9, 11	9, 11	1.0 ^e^
Neutered (yes, no)	19, 1	19, 1	1.0 ^e^
Breeds	Chihuahua (n = 8), Labrador Retriever mix (n = 4), Bichon Frise (n = 2), Miniature Pinscher (n = 2), Pomeranian (n = 2), Rottweiler (n = 2), Poodle mix (n = 2), Havanese (n = 2), Australian Shepherd mix (n = 2), Labrador retriever (n = 2), Jack Russel Terrier mix (n = 2), Miniature Pinscher mix (n = 2), Maltese–Poodle mix (n = 2), Rat Terrier (n = 2), Australian Cattle Dog (n = 2), Yorkshire Terrier (n = 2)		---

BCS, body condition score; kgs, kilograms; NODM, naturally occurring diabetes mellitus. ^a^ Data presented as mean (standard deviation). ^b^ Data presented as median (interquartile range). ^c^ Two-tailed Student’s *t*-test. ^d^ Mann–Whitney rank sum test. ^e^ Fisher’s exact test.

**Table 2:** Multivariable mixed-effects linear regression model of the association of diabetic status compared to controls, incubation in calcitriol compared to ethanol, and stimulation with LPS and LTA compared to PBS. n = 240, 240, 238, and 238 for IL-6, IL-8, IL-10, and TNF-α, respectively, after removal of outliers with Pearson residuals greater than 4.

	Variable	β	% Change	Robust Std. Error	z	*p*	95% CI			
**IL-6**	NODM	1.2	216%	0.17	6.8	<0.001	0.8	127%	1.5	340%
	Calcitriol	0.3	42%	0.13	2.8	0.005	0.1	11%	0.6	81%
	LPS	1.5	365%	0.13	11.7	<0.001	1.3	259%	1.8	502%
	LTA	1.3	274%	0.14	9.2	<0.001	1.0	182%	1.6	395%
**IL-8**	NODM	0.7	95%	0.17	3.9	<0.001	0.3	40%	1.0	172%
	Calcitriol	−0.1	−7%	0.04	−2.0	0.04	−0.1	−14%	0.0	0%
	LPS	0.5	61%	0.09	5.2	<0.001	0.3	35%	0.7	92%
	LTA	0.5	58%	0.09	4.9	<0.001	0.3	32%	0.6	90%
**IL-10**	NODM	0.5	60%	0.19	2.5	0.01	0.1	10%	0.8	133%
	Calcitriol	0.1	13%	0.08	1.6	0.11	0.0	−3%	0.3	32%
	LPS	2.5	1078%	0.15	17.0	<0.001	2.2	786%	2.8	1466%
	LTA	2.2	780%	0.15	14.1	<0.001	1.9	550%	2.5	1091%
**TNF-α**	NODM	0.4	50%	0.21	1.9	0.06	0.0	−1%	0.8	127%
	Calcitriol	−0.4	−33%	0.09	−4.5	<0.001	−0.6	−44%	−0.2	−20%
	LPS	2.2	760%	0.12	18.5	<0.001	1.9	585%	2.4	980%
	LTA	1.8	533%	0.12	15.7	<0.001	1.6	403%	2.1	697%

IL, interluekin; TNF, tumor necrosis factor; NODM, naturally occurring diabetes mellitus; LPS, lipopolysaccharide; LTA, lipoteichoic acid; std, standard.

## Data Availability

The data presented in this study are openly available in the Kaggle repository. https://www.kaggle.com/datasets/jaredjaffey/canine-nodm-immune-and-calcitriol accessed on 22 April 2024.

## Supplementary Materials

The following supporting information can be downloaded at: https://www.mdpi.com/article/10.3390/vetsci11050193/s1, Figure S1: Comparison of phagocytosis in samples incubated with calcitriol or ethanol; Figure S2: Comparison of phagocytosis in diabetic dogs that are clinically controlled and uncontrolled. Table S1: Descriptive data for each cytokine by group, intervention, and stimulant.
