# Supplementary material for: Ex Vivo Immune Function and Modulatory Effects of Calcitriol in Dogs with Naturally Occurring Diabetes Mellitus
Source: Vet Sci. 2024 Apr 28;11(5):193. doi: 10.3390/vetsci11050193 (PMC11125998; doi:10.3390/vetsci11050193)
Supplement: Supplementary file 1 [file vetsci-11-00193-s001.zip › vetsci-2909001-supplementary.pdf]

Supplemental Figure S1. Box and whisker plots illustrating (a) percentage of granulocytes and monocytes (GM) phagocytizing opsonized- *Escherichia coli* (*E. coli*) and (b) the mean fluorescent intensity (MFI) (i.e., the average number of *E. coli* phagocytized per cell) calcitriol and ethanol interventions, irrespective of group (naturally occurring diabetes mellitus [NODM] or non-diabetic control). Each of the 40 dogs, 20 per group (NODM or non-diabetic control), had a measure of phagocytosis for each intervention, for a total of 40 values per group for each plot. Line at median, bounds of box at the 25th and 75th percentile, whiskers at the upper and lower adjacent values (Tukey method), and dots at outliers beyond the adjacent values

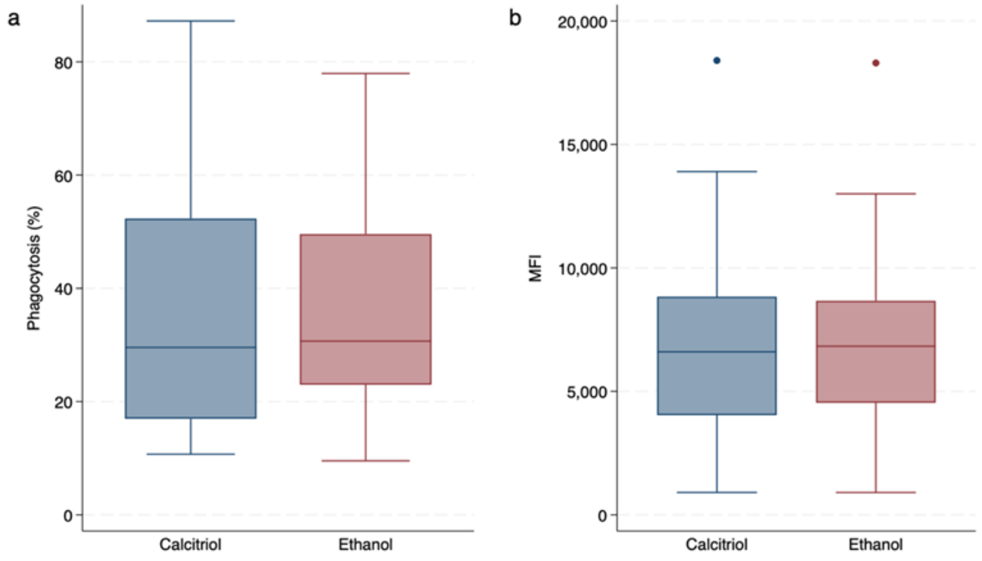

Supplemental Figure S2. Box and whisker plots illustrating (a) percentage of granulocytes and monocytes (GM) phagocytizing opsonized- *Escherichia coli* (*E. coli*) and (b) the mean fluorescent intensity (MFI) (i.e., the average number of *E. coli* phagocytized per cell) between dogs with controlled and uncontrolled naturally occurring diabetes mellitus (NODM), irrespective of diluent intervention (i.e., calcitriol or ethanol). Each of the 20 dogs, 10 per group (controlled or uncontrolled), had a measure of phagocytosis for each intervention, for a total of 20 values per group for each plot. Line at median, bounds of box at the 25th and 75th percentile, whiskers at the upper and lower adjacent values (Tukey method), and dots at outliers beyond the adjacent values.

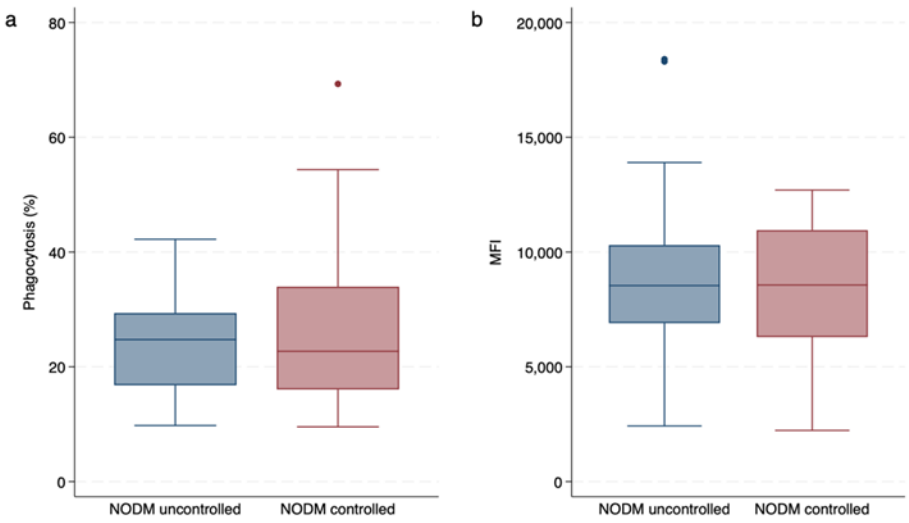

Supplemental Table S1. Median, interquartile range (IQR), and range for each cytokine by group (i.e., naturally occurring diabetic mellitus [NODM] or non-diabetic control; n = 20 per group), intervention, and stimulant.

Cytokine	Group	Intervention	Stimulant	Median	IQR	Range
**IL-6**	Control	Ethanol	LPS	87	49-147	49-256
			LTA	49	49-59	49-97
			PBS	49	49-49	49-104
		Calcitriol	LPS	71	51-167	49-348
			LTA	52	49-94	49-141
			PBS	49	49-49	49-101
	NODM	Ethanol	LPS	244	124-504	49-1,369
			LTA	168	101-317	49-1,086
			PBS	49	49-49	49-237
		Calcitriol	LPS	416	212-674	72-1,255
			LTA	318	202-695	108-1,231
			PBS	49	49-49	49-104
**IL-8**	Control	Ethanol	LPS	7,831	5,373-11,786	3,214-16,962
			LTA	8,489	5,748-11,485	834-17,746
			PBS	4,065	1,723-8,052	90-14,622
		Calcitriol	LPS	7,142	6,011-10,115	2,736-15,311
			LTA	7,485	5,059-10,286	2,557-18,442
			PBS	4,081	1,516-7,086	88-11,593
	NODM	Ethanol	LPS	14,910	9,115-20,260	4,944-38,460
			LTA	15,491	7,888-20,197	5,073-39,903
			PBS	12,637	5,459-19,013	4,138-28,650
		Calcitriol	LPS	15,739	7,345-21,597	5,275-50,122

			LTA	14,811	8,348-21479	5,064-33,342
			PBS	10,231	5,706-13,603	1,430-34,463
**IL-10**	Control	Ethanol	LPS	1,965	1,438-3,559	693-9,044
			LTA	831	456-1,794	132-3,839
			PBS	91	49-199	49-6,737
		Calcitriol	LPS	2,122	910-3,301	373-8,412
			LTA	1,052	667-2,518	201-3,716
			PBS	180	79-500	49-2,297
	NODM	Ethanol	LPS	2,286	1,716-3,792	739-8,170
			LTA	2,400	2,072-3,524	420-6,349
			PBS	154	450-293	49-849
		Calcitriol	LPS	2,429	1,375-4,102	519-4,878
			LTA	2,688	1,807-3,642	508-6,598
			PBS	211	52-593	49-1,956
**TNF-α**	Control	Ethanol	LPS	534	341-1,641	204-6,078
			LTA	440	249-649	128-4,033
			PBS	95	49-235	49-6,981
		Calcitriol	LPS	290	207-1,037	75-3,948
			LTA	268	144-453	70-2,113
			PBS	51	49-96	49-2706
	NODM	Ethanol	LPS	1,260	670-1,521	419-5,048
			LTA	933	728-1,188	305-3,510
			PBS	88	58-164	49-554
		Calcitriol	LPS	504	617-1,560	159-3,361
			LTA	753	471-1,395	232-2,414
			PBS	49	49-83	49-192
